# Analysis of Anticoagulation Therapy and Anticoagulation-Related Outcomes Among Asian Patients After Mechanical Valve Replacement

**DOI:** 10.1001/jamanetworkopen.2021.46026

**Published:** 2022-02-01

**Authors:** Jo-Ting Huang, Yi-Hsin Chan, Victor Chien-Chia Wu, Yu-Ting Cheng, Dong-Yi Chen, Chia-Pin Lin, Kuo-Chun Hung, Shang-Hung Chang, Pao-Hsien Chu, An-Hsun Chou, Shao-Wei Chen

**Affiliations:** 1 Department of Education, Chang Gung Memorial Hospital, Chang Gung University College of Medicine, Taoyuan City, Taiwan; 2 Department of Cardiology, Chang Gung Memorial Hospital, Linkou Medical Center, Chang Gung University, Taoyuan City, Taiwan; 3 Division of Thoracic and Cardiovascular Surgery, Department of Surgery, Chang Gung Memorial Hospital, Linkou Medical Center, Chang Gung University, Taoyuan City, Taiwan; 4 Center for Big Data Analytics and Statistics, Chang Gung Memorial Hospital, Linkou Medical Center, Taoyuan City, Taiwan; 5 Department of Anesthesiology, Chang Gung Memorial Hospital, Linkou Medical Center, Chang Gung University, Taoyuan City, Taiwan

## Abstract

**Importance:**

Current international normalized ratio (INR) guidelines are based on trials involving European and US populations. To our knowledge, no adequate study involving Asian patients has been conducted to date.

**Objective:**

To evaluate the association between INR and anticoagulation-related outcomes in an Asian population after mechanical aortic valve replacement (AVR) or mitral VR (MVR).

**Design, Setting, and Participants:**

This retrospective cohort study was conducted between 2001 and 2018, with follow-up until December 31, 2018, among patients who underwent AVR, MVR, or combined AVR-MVR at 3 medical centers and 4 regional hospitals and contributed electronic medical records to the Chang Gung Research Database. Exclusion criteria were missing demographic characteristics, younger than 20 years, fewer than 2 INR records, and having died during the hospitalization of the index surgery.

**Main Outcomes and Measures:**

Bleeding and thromboembolic complications were analyzed. The possibility of nonlinearity and cutoff potential for the INR were explored using a logistic regression model, which considered the INR a restricted cubic spline (RCS) variable.

**Results:**

The study population consisted of 900 patients, with 525 (58.3%) men and 375 (41.7%) women and a mean (SD) age of 52.0 (12.5) years. Overall, 474 (52.7%) received AVR alone, 329 (36.6%) received MVR alone, and 97 (10.8%) received combined AVR-MVR. All patients had at least 2 INR examinations after discharge, providing 16 676 INR records for the AVR group and 18 207 for the MVR and combined AVR-MVR groups. In the AVR group, the RCS model showed that higher risks of composite thromboembolic events were associated with an INR of less than 2.0 or greater than 2.6 vs an INR of 2.0, and a higher risk of bleeding events was associated with an INR of less than 1.8 or greater than 2.4 vs an INR of 2.0. When treating the INR as a categorical variable, the risk of composite thromboembolic and composite bleeding events was significantly higher among patients with INRs less than 1.5 (adjusted odds ratio [aOR], 2.55; 95% CI, 1.37-4.73) and with INRs of 3.0 or greater (aOR, 3.48; 95% CI, 1.95-6.23) vs those with INRs between 2.0 and 2.5.In the MVR and combined AVR-MVR groups, higher risks of composite thromboembolic events were associated with an INR of less than 2.1 or greater than 2.7 vs an INR of 2.5, and a higher risk of bleeding events was associated with an INR of less than 2.1 or greater than 2.8 vs an INR of 2.5. When treating the INR as a categorical variable, the risk of a composite bleeding events was significantly higher among patients with INRs of 3.5 or greater (aOR, 2.25; 95% CI, 1.35-3.76) vs those with INRs between 2.5 and 3.0.

**Conclusions and Relevance:**

Among Asian patients in this study, the incidence of thromboembolic events in the MVR group with INRs in the range of 2.0 to 2.5 was not significantly higher than that among those with INRs in the range of 2.5 to 3.0; in the AVR group, the incidence for those with INRs in 1.5 to 2.0 range was not significantly higher than for those with INRs in the range of 2.0 to 2.5.

## Key Points

**Question:**

Is a low international normalized ratio (INR) target associated with a low rate of thromboembolic complications among Asian patients with mechanical valve replacement?

**Findings:**

This cohort study included 900 patients who underwent mechanical valve replacement, providing 34 883 INR records. In the MVR group, the incidence of thromboembolic events among patients with INRs in the range of 2.0 to 2.5 was not significantly higher than that among those with INRs in the range of 2.5 to 3.0; in the AVR group, the incidence among patients with INRs in the range of 1.5 to 2.0 was not significantly higher than that among those with INRs in the range of 2.0 to 2.5.

**Meaning:**

The findings of this study suggest that a lower INR range may not be associated with increased risk of thromboembolic events in Asian populations.

## Teaser

This cohort study evaluates the association between international normalized ratio (INR) and rate of anticoagulation-related outcomes in an Asian population after mechanical aortic and/or mitral valve replacement.

## Introduction

Valvular heart disease is a common cardiovascular condition, comprising more than 10% of all cardiac surgical procedures in the United States. According to the 2020 American Heart Association/American College of Cardiology (AHA/ACC) guideline, prosthetic valve replacement is recommended when symptoms develop or valve repair is inappropriate.^1,2,3^ Tissue or mechanical valves can be used for heart valve replacement. For both mitral valve replacement (MVR) and aortic valve replacement (AVR), mechanical valves have resulted in a long-term mortality benefit in young patients due to the considerably lower reoperation risk compared with tissue valves.^4,5^ Patients who receive mechanical valve replacement surgery must take anticoagulants for the rest of their lives, mostly warfarin.

Novel oral anticoagulants (NOACs) are considered an alternative to warfarin in mechanical valve replacement. So far, 2 randomized clinical trials have compared NOAC and warfarin, ie, the Randomized Phase 2 Study to Evaluate the Safety and Pharmacokinetics of Oral Dabigatran Etexilate in Patients after Heart Valve Replacement (RE-ALIGN) and the Rivaroxaban vs Warfarin in Patients With Metallic Prosthesis (RIWA) studies. The RE-ALIGN study found that dabigatran was not as effective as warfarin at the tested dose for thromboembolic event prevention after mechanical valve replacement, and furthermore, it had a higher bleeding risk.^6^ However, it has some limitations, such as an inadequate trough plasma level and different mechanism from other NOACs. In the RIWA study, 15 mg of rivaroxaban twice daily exhibited similar numbers of bleeding and thromboembolic events compared with warfarin.^7^ However, contrary to the aforementioned study, thrombolytic^8^ and even fatal^9^ outcomes have been described. Furthermore, apixaban demonstrated comparable efficacy in preclinical AVR models.^10^ Further investigations are warranted; however, warfarin remains the first-choice anticoagulant after mechanical valve surgery.

To balance the thromboembolic and bleeding risk, regular monitoring of international normalized ratio (INR) is indispensable during warfarin therapy. Factors such as drug and food interaction, age, sex, and race may all affect bleeding risk. Obtaining an optimal INR can prevent complications. According to the 2021 European Society of Cardiology/European Association for Cardio-Thoracic Surgery (ESC/EACTS) and 2020 AHA/ACC guidelines, the INR targets for AVR and MVR are 2.5 and 3.0 respectively. All current guidelines are based on trials involving European and US populations. However, the clinical INR range for Europeans and US residents is considerably higher than that for the Asian population.^11^ Various studies have also reported that anticoagulant requirements vary according to racial background,^12,13^ but precise studies are still lacking. We hypothesized that for an Asian population, an INR target lower than that recommended in current guidelines is preferable.

Thus, this study investigated the association between INR range and the incidence of bleeding and thromboembolism by using a multicenter medical database. The objective of this study was to examine the optimal INR target for an Asian population after mechanical AVR or MVR.

## Methods

### Data Source

This retrospective cohort study was conducted using the Chang Gung Research Database (CGRD), a deidentified database based on the electronic medical records of the Chang Gung Memorial Hospital (CGMH), one of the biggest health care networks in east Asia. It provides medical services to more than 1 million outpatients and 0.3 million inpatients annually. In the CGRD, diagnosis, medical orders, imaging, laboratory examinations, medications, and procedures are detailed, with personal medical information encrypted and available for research purposes. Diseases are identified using *International Classification of Diseases, Ninth Revision, Clinical Modification* (*ICD-9-CM*) diagnostic codes for records before 2015 and *International Statistical Classification of Diseases, Tenth Revision* (*ICD-10-CM*) diagnostic codes for those after 2016. Detailed information on the CGRD is published elsewhere.^14^ Informed consent was waived by the institutional review board of the CGMH because personal medication information was encrypted. This study followed the Strengthening the Reporting of Observational Studies in Epidemiology (STROBE) reporting guideline.

### Patient Identification

**Figure 1.**
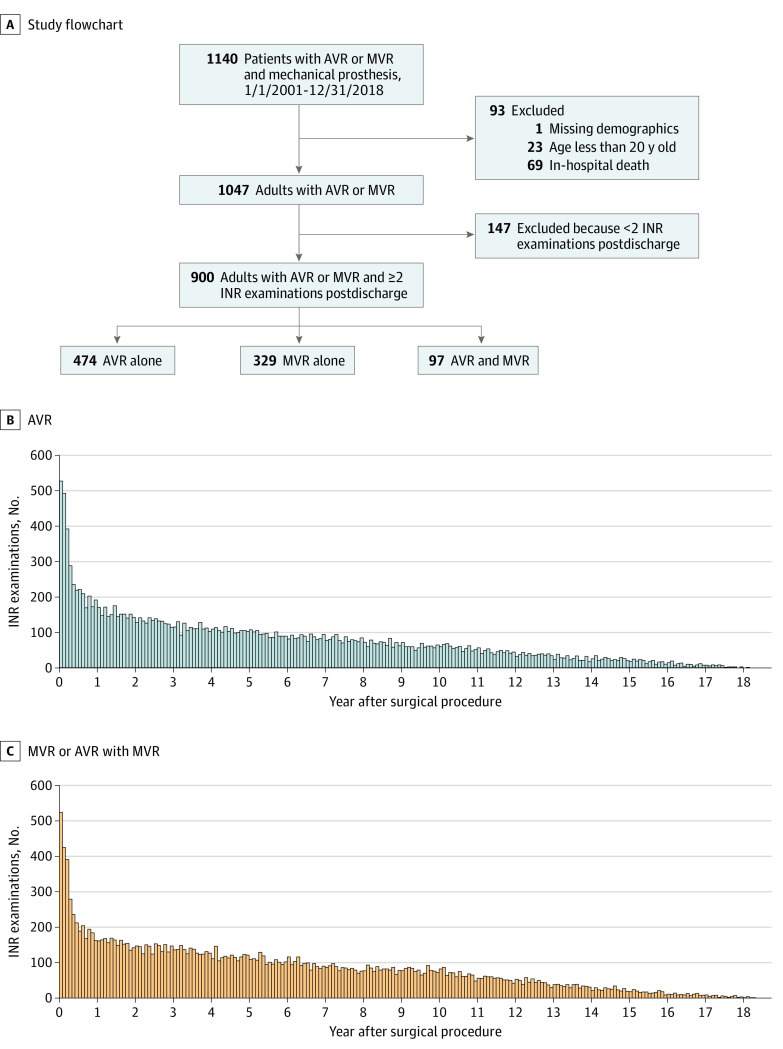
Study Flowchart and the Number of International Normalized Ratio (INR) Examinations After Aortic Valve Replacement (AVR) and Mitral Valve Replacement (MVR) or Combined MVR-AVR Operations

Patients who received AVR and/or MVR with mechanical prosthesis from January 1, 2001, to December 31, 2018, were included. The first valve replacement the patient received during the study period was chosen as the index surgery if the patient received 2 or more valve replacements. The valve replacement surgery was ascertained by combining Taiwan National Health Insurance (NHI) reimbursement codes (for valve replacement performed), *ICD-9-CM* or *ICD-10-CM* procedural codes, and Taiwan NHI reimbursement codes of supplies (the implanted valve). Patients were categorized into the AVR alone, MVR alone, and combined AVR-MVR groups. Patients with missing demographic information, those younger than 20 years, and those who died during the hospitalization of the index surgery were excluded. Furthermore, patients with fewer than 2 INR records were excluded (Figure 1).

### Data Unit

The index date was the discharge date after hospitalization for the index surgery. The data unit was from a prior INR examination to the next one. For instance, 30 rows of data were present if the patient had 31 INR examinations after discharge. The first unit of data for the patient was from the first INR examination after discharge to the second one. Every point and INR after discharge were recorded. If multiple INR records for the same emergency department visit or admission were recorded, only the first INR record was included. By contrast, all INR records from outpatient visits were retained. The patient follow-up was from the last examination date of the INR to the latest visit date in the CGRD, and even those who died in the hospital were included.

### Outcomes

This study had 2 primary outcomes: a composite bleeding event and a composite thromboembolic event. The outcome occurrence was captured 1 day before to 7 days after the date of each INR examination.^15^ The composite thromboembolic event consisted of ischemic stroke, acute myocardial infarction, systemic thromboembolism, or bowel ischemia. The composite bleeding event was hemorrhagic stroke, gastrointestinal bleeding, genitourinary bleeding, and major bleeding (which had a broad definition). Event occurrence was defined by a discharge diagnosis for an emergency department visit or hospitalization. Multiple episodes of an outcome (ie, composite thromboembolic events) for one patient were allowed during follow-up visits. However, an outcome occurrence for each INR examination was counted only once.

### Covariates

Covariates were age, sex, height, body weight, body surface area, smoking, left ventricular ejection fraction (LVEF), comorbid conditions, medications, and concomitant surgeries (tricuspid valve surgery and maze). Comorbidities, event history, and medications were rechecked during each INR examination. Comorbidities were defined as at least 2 outpatient diagnoses or at least 1 inpatient diagnosis before each INR examination. Event history was defined as at least 1 inpatient diagnosis before each INR examination. Medication data were extracted using the records for the 3 months before each INR examination.

### Statistical Analysis

The AVR-alone group was analyzed separately from the MVR-alone and MVR-AVR–combination groups. During the analysis of the association between INR and bleeding or thromboembolic event risk, a generalized estimating equation (GEE) logistic model with exchangeable working correlation structure was used to account for the within-patient correlation among multiple data units of the patient. INRs were presented as a continuous variable or a categorized variable in separate models. The reference category of INR was 2.0 to 2.5 among the AVR-alone group and 2.5 to 3.0 among the MVR-alone and combined MVR-AVR surgery groups.

The continuous INR was further treated as a restricted cubic spline (RCS) variable in the logistic regression model. Nonlinearity possibility and cutoff potential for the INR were explored in the RCS model. Four knots were located at 5th, 35th, 65th, and 95th percentiles. The within-patient correlation was accounted for by using bootstrap estimates through the substitution of cluster sampling with replacement for the usual simple sampling with replacement. In total, 200 bootstrap samples were used.

All covariates mentioned previously and listed in Table 1 were adjusted in both GEE and RCS models. Because data on height, body weight, body surface area, and LVEF were missing, a single expectation maximization imputation was performed before covariate adjustment. RCS modeling was performed with R version 4.0.2 (R Project for Statistical Computing) and the package rms version 5.1 to 3.1. Other statistical analyses were performed using SAS version 9.4 (SAS Institute). A 2-sided *P* < .05 was considered statistically significant.

**Table 1.** Basic Patient Demographic Characteristics

Characteristic	Patients with valid data, No.	Patients, No. (%)			
		Total (N = 900)	AVR alone (n = 474)	MVR alone (n = 329)	AVR and MVR (n = 97)
Age, mean (SD), y	900	52.0 (12.5)	52.8 (14.1)	51.8 (10.6)	49.3 (9.7)
Male sex	900	525 (58.3)	310 (65.4)	159 (48.3)	56 (57.7)
Female sex	900	375 (41.7)	164 (34.6)	170 (51.7)	41 (42.3)
Height, mean (SD), cm	674	162.4 (10.3)	163.1 (11.6)	161.4 (8.9)	162.6 (7.8)
Body weight, mean (SD), kg	720	62.4 (14.4)	64.3 (15.7)	60.5 (12.7)	59.2 (11.0)
Body surface area, mean (SD), m^2^	669	1.67 (0.21)	1.69 (0.23)	1.64 (0.19)	1.64 (0.18)
Smoking	900	188 (20.9)	117 (24.7)	55 (16.7)	16 (16.5)
Comorbid conditions					
Atrial fibrillation	900	368 (40.9)	84 (17.7)	233 (70.8)	51 (52.6)
COPD	900	112 (12.4)	49 (10.3)	52 (15.8)	11 (11.3)
Chronic liver disease	900	162 (18.0)	74 (15.6)	71 (21.6)	17 (17.5)
Chronic kidney disease	900	114 (12.7)	61 (12.9)	41 (12.5)	12 (12.4)
Hypertension	900	336 (37.3)	202 (42.6)	110 (33.4)	24 (24.7)
Hyperlipidemia	900	176 (19.6)	100 (21.1)	64 (19.5)	12 (12.4)
Diabetes	900	130 (14.4)	71 (15.0)	48 (14.6)	11 (11.3)
Prior ischemic stroke	900	62 (6.9)	32 (6.8)	23 (7.0)	7 (7.2)
Prior myocardial infarction	900	19 (2.1)	10 (2.1)	7 (2.1)	2 (2.1)
Infective endocarditis	900	139 (15.4)	66 (13.9)	45 (13.7)	28 (28.9)
Rheumatic heart disease	900	396 (44.0)	134 (28.3)	202 (61.4)	60 (61.9)
Lung edema	900	70 (7.8)	33 (7.0)	31 (9.4)	6 (6.2)
LVEF, mean (SD), %	772	59.8 (13.8)	59.1 (14.8)	60.8 (12.4)	60.3 (13.5)
History of gastrointestinal bleeding	900	79 (8.8)	39 (8.2)	31 (9.4)	9 (9.3)
History of intracranial Hemorrhage	900	39 (4.3)	32 (6.8)	3 (0.91)	4 (4.1)
History of major bleeding	900	30 (3.3)	15 (3.2)	11 (3.3)	4 (4.1)
Gout	900	106 (11.8)	56 (11.8)	40 (12.2)	10 (10.3)
Peripheral artery disease	900	185 (20.6)	152 (32.1)	23 (7.0)	10 (10.3)
Malignant neoplasm	900	34 (3.8)	25 (5.3)	8 (2.4)	1 (1.03)
Concurrent medications					
Statins	900	70 (7.8)	44 (9.3)	20 (6.1)	6 (6.2)
Antiplatelet	900	174 (19.3)	114 (24.1)	51 (15.5)	9 (9.3)
Amiodarone	900	174 (19.3)	83 (17.5)	76 (23.1)	15 (15.5)
β-blocker	900	380 (42.2)	222 (46.8)	126 (38.3)	32 (33.0)
AECi/ARB	900	440 (48.9)	239 (50.4)	153 (46.5)	48 (49.5)
NSAIDs	900	117 (13.0)	65 (13.7)	43 (13.1)	9 (9.3)
Proton pump inhibitor	900	75 (8.3)	44 (9.3)	25 (7.6)	6 (6.2)
Concomitant surgery					
Tricuspid valve surgery	900	83 (9.2)	17 (3.6)	53 (16.1)	13 (13.4)
Maze	900	123 (13.7)	19 (4.0)	82 (24.9)	22 (22.7)

Abbreviations: ACEi, angiotensin converting enzyme inhibitor; ARB, angiotensin receptor blocker; AVR, aortic valve replacement; COPD, chronic obstructive pulmonary disease; LVEF, left ventricular ejection fraction; MVR, mitral valve replacement; NSAIDs, nonsteroidal anti-inflammatory drugs.

## Results

### Baseline Data

The mean (SD) age of the 900 adult patients was 52 (12.5) years; 525 (58.3%) were men, and 375 (41.7%) were women. Overall, 474 patients (52.7%) received MVR alone; 329 (36.6%), AVR alone, and 97 (10.8%), a combination of AVR and MVR (Figure 1A). The demographic and clinical characteristics at baseline are listed in Table 1. The patients were followed up from the first INR examination after discharge of the index surgery, providing 16 676 INR records for the AVR-alone group (Figure 1B) and 18 207 INR records for the MVR-alone and combined AVR-MVR groups (Figure 1C). The mean INR level was stable during the long period of 2001 to 2018 in all the patient groups (eFigure in the Supplement).

### Follow-up Information

The follow-up information after discharge following the index surgery is presented in Table 2. The mean (SD) INRs at discharge were 1.79 (0.54), 2.03 (0.61), and 2.05 (0.62) in the AVR-alone, MVR-alone, and AVR-MVR–combination groups, respectively. During the follow-up, which was a mean (SD) of 7.6 (5.2) years in the AVR-alone group and 7.7 (5.2) years and MVR-alone group, the mean (SD) INR was 1.87 (0.41) and 2.17 (0.39), with a mean (SD) of 35 (31) and 42 (33) INR records per patient, respectively. Thromboembolic event rates in the AVR-alone and MVR-alone groups were 13.1% (62 patients) and 14.3% (47 patients), respectively, with ischemic stroke being the most prevalent thromboembolic event. Regarding bleeding events, the event rates in the AVR-alone and MVR-alone group were 22.8% (108 patients) and 24.6% (81 patients), respectively.

**Table 2.** Data and Events of Interest During Follow-up

Variable	Patients, No. (%)^a^			
	Total (N = 900)	AVR alone (n = 474)	MVR alone (n = 329)	AVR and MVR (n = 97)
Follow-up information, mean (SD)				
INR at discharge	1.91 (0.58)	1.79 (0.54)	2.03 (0.61)	2.05 (0.62)
Baseline INR	2.04 (0.94)	1.82 (0.76)	2.29 (1.10)	2.26 (0.90)
Last INR	2.09 (0.96)	1.95 (0.81)	2.22 (1.12)	2.34 (0.93)
INR during follow-up	2.02 (0.43)	1.87 (0.41)	2.17 (0.39)	2.21 (0.38)
INR examinations per patient, No.	39 (33)	35 (31)	42 (33)	46 (36)
Follow-up duration, y	7.7 (5.2)	7.6 (5.2)	7.7 (5.2)	8.0 (5.1)
During follow-up				
Thromboembolic events				
Ischemic stroke	84 (9.3)	42 (8.9)	37 (11.3)	5 (5.2)
Acute myocardial infarction	30 (3.3)	18 (3.8)	10 (3.0)	2 (2.1)
Systemic thromboembolism	16 (1.8)	8 (1.7)	7 (2.1)	1 (1.03)
Lower extremity systemic thromboembolism	10 (1.1)	4 (0.84)	6 (1.8)	0 (0.0)
Ischemia bowel	4 (0.44)	3 (0.63)	0 (0.0)	1 (1.03)
Any other thromboembolic events	117 (13.0)	62 (13.1)	47 (14.3)	8 (8.3)
Total bleeding events				
Hemorrhagic stroke	41 (4.6)	21 (4.4)	15 (4.6)	5 (5.2)
Gastrointestinal bleeding	147 (16.3)	81 (17.1)	54 (16.4)	12 (12.4)
Genitourinary bleeding	47 (5.2)	13 (2.7)	22 (6.7)	12 (12.4)
Major bleeding	109 (12.1)	60 (12.7)	39 (11.9)	10 (10.3)
Any other bleeding events	215 (23.9)	108 (22.8)	81 (24.6)	26 (26.8)

Abbreviations: AVR, aortic valve replacement; INR, international normalized ratio; MVR, mitral valve replacement.

^a^ All 900 patients had valid data.

### Association Between INR and Outcomes in the AVR Group

**Figure 2.**
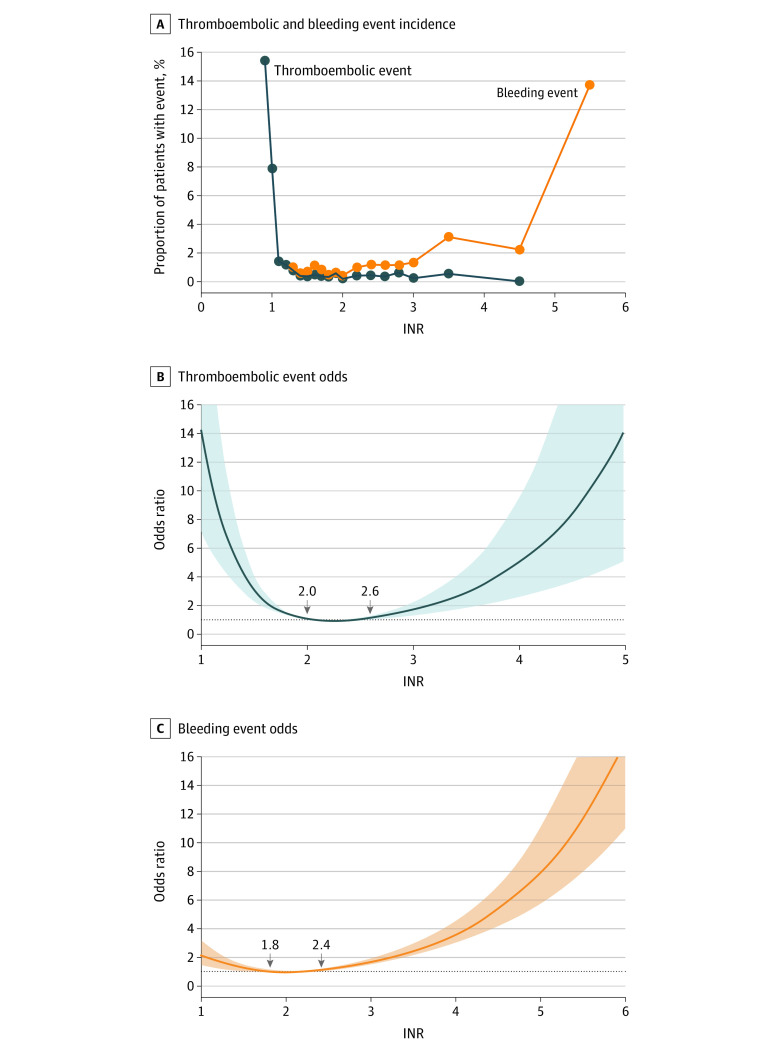
Association of the International Normalized Ratio (INR) With Incidence of Thromboembolic and Bleeding Events and Nonlinear Associations of the INR With Thromboembolic Event Risk and Bleeding Event Risk in Patients Receiving Aortic Valve Replacement Surgery A, Dots indicate the grouped INR data. B and C, The shaded areas indicate 95% CIs. The reference level for the INR was 2.0.

In the AVR-alone group, an extremely low INR (ie, <1.5) was associated with a high risk of a composite thromboembolic event. Furthermore, a high INR level was associated with a high risk of a composite bleeding event, particularly when the INR was greater than 2 (Figure 2A). When treating the INR as a categorical variable, the risk of composite thromboembolic and composite bleeding events was significantly higher in the lowest INR range of less than 1.5 (adjusted odds ratio [aOR], 2.55; 95% CI, 1.37-4.73) and in the highest INR range of 3.0 or greater (aOR, 3.48; 95% CI, 1.95-6.23) compared with the reference category of an INR between 2.0 and 2.5 (eTable 1 in the Supplement). Compared with an INR of 2.0, the RCS model showed that an INR of less than 2.0 or greater than 2.6 was associated with a higher risk of composite thromboembolic event (Figure 2B). Conversely, an INR of less than 1.8 or greater than 2.4 was associated with a high risk of a composite bleeding event vs an INR of 2.0 (Figure 2C).

### Association Between INR and Outcomes in the MVR-Alone and Combined Groups

**Figure 3.**
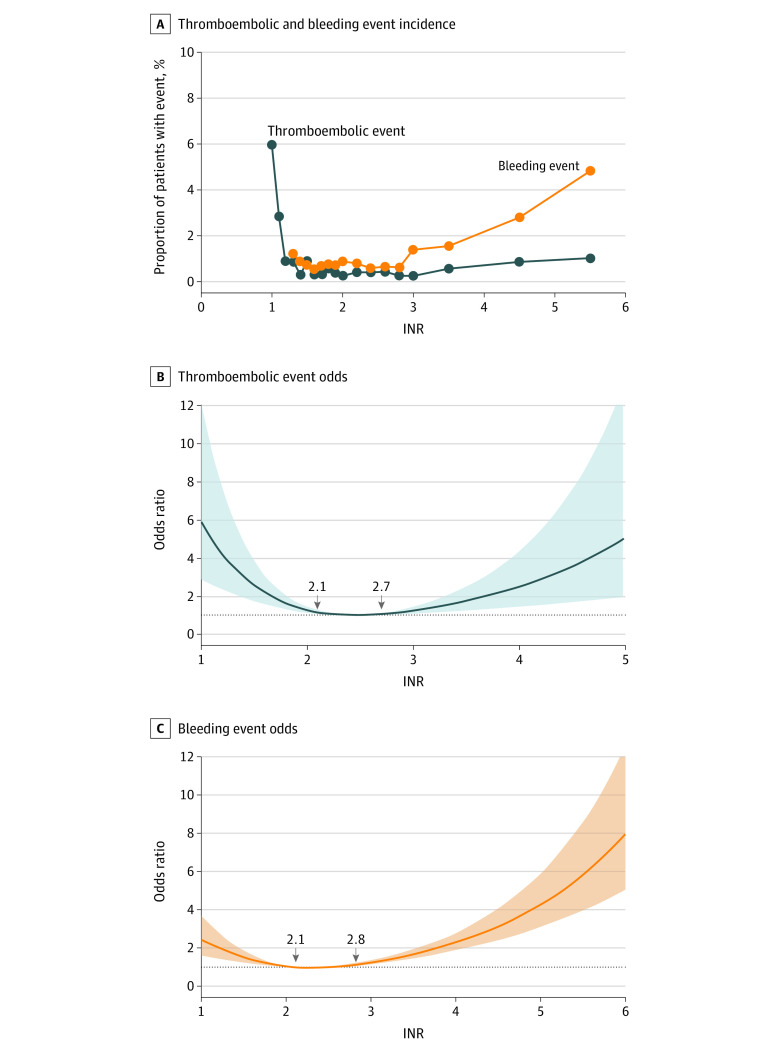
Association of the International Normalized Ratio (INR) With Incidences of Thromboembolic and Bleeding Events and Nonlinear Associations of the INR With Thromboembolic Event Risk and Bleeding Event Risk in Patients Receiving Mitral Valve Replacement or Combined Mitral Valve Replacement–Atrial Valve Replacement Surgery A, Dots indicate the grouped INR data. B and C, The shaded areas indicate 95% CIs. The reference level for the INR was 2.0.

In the MVR-alone and MVR-AVR–combination groups, an extremely low INR level (ie, <1.5) was associated with a high risk of a composite thromboembolic event. Furthermore, a high INR level was associated with a high risk of a composite bleeding event, particularly when the INR was greater than 3 (Figure 3B). The risk of a composite bleeding event was significantly greater when the INR was at its highest value, 3.5 or greater (aOR, 2.25; 95% CI, 1.35-3.76) vs the reference INR ranging from 2.5 to 3.0. However, no association was observed between the INR and risk of a composite thromboembolic event (eTable 2 in the Supplement). Compared with an INR of 2.5, the RCS model revealed that an INR of less than 2.1 or greater than 2.7 was associated with a higher risk of composite thromboembolic event (Figure 3B). Conversely, an INR of less than 2.1 or greater than 2.8 was associated with a high risk of a composite bleeding event compared with an INR of 2.5 (Figure 3C).

## Discussion

To our knowledge, this is the largest in-hospital cohort study of the INR target for mechanical valve replacement in Asia. It was based on a multicenter medical database containing surgery details as well as laboratory and medication data, providing more than 30 000 INR records. The incidence of thromboembolic events in the MVR group with INRs in the range of 2.0 to 2.5 was not significantly higher than that for those with INRs in the 2.5 to 3.0 range; in the AVR group, the incidence of thromboembolic events for those with INRs in 1.5 to 2.0 range was not significantly higher than that for those with INRs in the 2.0 to 2.5 range.

In the early 1990s, an INR target of 3.0 to 4.5 was recommended for preventing thromboembolic events after mechanical heart valve replacement.^16^ However, a lower level of anticoagulation was suggested because of less bleeding with no increase in thromboembolism. By then, different implantation sites or prosthesis types were not considered when analyzing the optimal INR. Gohlke-Bärwolf et al^17^ suggested an INR target of 2.5 to 3.0 after AVR and 3.0 to 3.5 after MVR. The Multicenter Randomized Comparison of Low-Dose vs Standard-Dose Anticoagulation in Patients With Mechanical Prosthetic Heart Valves (AREVA) trial^18^ reported that the thromboembolic incidence of an INR target between 2.0 and 3.0 was comparable with that between 3.0 and 4.5, but the former was associated with fewer bleeding events. In the Lowering the Intensity of Oral Anticoagulant Therapy in Patients With Bileaflet Mechanical Aortic Valve Replacement (LOWERING-IT) trial,^19^ the thromboembolic events in the low INR group (an INR target of 1.5-2.5) was noninferior to the conventional INR group (an INR target of 2.0-3.0), but bleeding events in the low INR group were statistically decreased. The 2 aforementioned prospective studies^18,19^ mainly investigated patients who underwent AVR. The randomized German Experience with Low Intensity Anticoagulation study^20,21^ suggested an INR target of 2.0 to 3.5 for MVR and 2.5 to 4.0 for combined valve replacement when using a St Jude mechanical bileaflet prosthetic valve.

However, Asian individuals are more sensitive and vulnerable to warfarin, which leads to a lower maintenance dose requirement and higher hemorrhagic risk than US and European populations.^12,13^ Studies have indicated that the INR target for atrial fibrillation in Asian individuals should be lower than that suggested by Western guidelines.^22,23^ A meta-analysis of East Asian patients with nonvalvular atrial fibrillation receiving warfarin therapy indicated that the an INR of 1.5 to 2.5 is the most appropriate considering both thromboembolism and hemorrhage.^24^ Because the event risk of mechanical valve replacement was different from that of atrial fibrillation, knowing the optimal INR range for Asian patients after mechanical valve replacement is vital.

A few retrospective cohort studies have attempted to determine a favorable INR target. Mori et al^25^ enrolled 102 Japanese patients who had mechanical valve replacement. In total, 1846 INR records were available with a mean (SD) of 7.7 (2.3) records per year per patient, and the follow-up duration was 2 years and 5 months. The complications were associated with a specific INR record when the INR was recorded within 2 weeks before and after the event. An INR of less than 2.5 was associated with fewer bleeding events without increased risk of thromboembolism.^25^ To analyze warfarin-induced event rates, You et al^15^ followed 491 Chinese patients who newly received warfarin with an INR target of 2.0 to 3.0 for various indications, mostly atrial fibrillation. A major event was included if an INR was recorded at admission or in the outpatient department less than 7 days before the event. The INR range of 1.8 to 2.4 was associated with the least hemorrhage and thromboembolism.^15^ Yu et al^26^ from National Taiwan University Hospital recruited 563 Taiwanese patients who took warfarin with an INR target of 1.5 to 2.5 after mechanical AVR or MVR from 1996 to 2001. The follow-up periods were grouped into several 6-month intervals. In case of bleeding or thromboembolic events, the nearest INR within 6 months before the event was recorded. They concluded that an INR of less than 2.0 was not associated with higher thromboembolism or lower bleeding rates for patients in their sample.^26^ Based on these studies, a lower INR target compared with current Western guidelines could decrease bleeding events without increasing thromboembolism risk. However, the study population and number of INR records were all small, and they did not suggest a precise INR target for mechanical valve replacement. Compared with previous Asian studies, our study included, to our knowledge, the largest patient numbers and INR records and concluded that the incidence of thromboembolic events among patients in the MVR group with an INR in the range of 2.0 to 2.5 was not significantly higher than that for those with an INR in the 2.5 to 3.0 range; among patients in the AVR group, the incidence of thromboembolic events among patients with an INR in 1.5 to 2.0 range was not significantly higher that among those with an INR in the 2.0 to 2.5 range.

Because the United States is a multiracial country, with 6.6% of its population consisting of Asian individuals in 2019 according to the US Census Bureau, the association of racial difference with anticoagulant intensity requirements should be determined. Further studies on anticoagulant use, including the INR target in various racial and ethnic populations as well as new types of mechanical valves or NOACs, will lead to better long-term outcomes of mechanical valve replacement.

### Limitations

Our study had several limitations. First, we used *ICD-9* and *ICD-10* procedure codes to classify the locations of valve surgery (AVR and MVR), which may cause bias owing to coding error. However, we additionally used supply codes relating to the implanted valve to ascertain the valve surgery location. Through examination of the operation notes, our internal validation showed that claim data accuracy from operation records was as high as 98% (data not shown). In addition, the use of *ICD-9-CM* and *ICD-10-CM* codes to identify bleeding and thromboembolic events was subject to potential coding errors and inconsistencies. Positive predictive values for acute myocardial infarction,^27^ acute ischemic stroke,^28^ and intracerebral hemorrhage^29^ have been greater than 90% using the claims data. Second, in our medical system, not all medical valve types were available during the study period. In this study, both the St Jude mechanical valve and On-X valve were used. However, the INR target of the On-X valve is lower than that of the St Jude mechanical valve,^30^ which does not contradict our conclusion but may require further studies to determine the optimal target. Furthermore, retrospective studies have inherent limitations, and retrospective database studies have associated biases. We could investigate associations but could not infer causation. The generalizability may be limited because data came from a single medical system. However, given that CGMH is among the largest health care systems in east Asia, the representativeness of the study should be recognized. We believe that this research contributes to the field, and we expect further studies to verify our results.

## Conclusions

In this study, the incidence of thromboembolic events among patients in the MVR group with INRs in the range of 2.0 to 2.5 was not significantly higher than that among those with INRs in the 2.5 to 3.0 range; in the AVR group, the incidence of thromboembolic events among patients with INRs in 1.5 to 2.0 range was not significantly higher than that among those with INRs in the 2.0 to 2.5 range. Further randomized clinical trials are warranted to develop a therapeutic recommendation for the Asian population.

## Notes

Supplement. **eFigure.** Average INR Value Across 2001 to 2018 in Patients With Isolated Aortic Valve Replacement and Mitral Valve Replacement or Double Valve Replacement **eTable 1. **Association Between INR Value and Risk of Thromboembolic and Bleeding Events in Patients Undergoing AVR Surgery **eTable 2. **Association Between INR and Risks of Thromboembolic and Bleeding Events in Patients Receiving MVR Alone or MVR-AVR Combination Surgery

## References

[1] Wang Z, Zhou C, Gu H, Zheng Z, Hu S. Mitral valve repair versus replacement in patients with rheumatic heart disease. J Heart Valve Dis. 2013;22(3):333-339. 24151759

[2] Lee HA, Cheng YT, Wu VC, . Nationwide cohort study of mitral valve repair versus replacement for infective endocarditis. J Thorac Cardiovasc Surg. 2018;156(4):1473-1483.e2. doi:10.1016/j.jtcvs.2018.04.064 29843917

[3] Wong WK, Chen SW, Chou AH, . Late outcomes of valve repair versus replacement in isolated and concomitant tricuspid valve surgery: a nationwide cohort study. J Am Heart Assoc. 2020;9(8):e015637. doi:10.1161/JAHA.119.015637 32301369 PMC7428522

[4] Goldstone AB, Chiu P, Baiocchi M, . Mechanical or biologic prostheses for aortic-valve and mitral-valve replacement. N Engl J Med. 2017;377(19):1847-1857. doi:10.1056/NEJMoa1613792 29117490 PMC9856242

[5] Chen SW, Wu VC, Lin YS, . Propensity score matched analysis of mechanical vs. bioprosthetic valve replacement in patients with previous stroke. Circ J. 2018;82(8):2041-2048. doi:10.1253/circj.CJ-18-0003 29794401

[6] Eikelboom JW, Connolly SJ, Brueckmann M, ; RE-ALIGN Investigators. Dabigatran versus warfarin in patients with mechanical heart valves. N Engl J Med. 2013;369(13):1206-1214. doi:10.1056/NEJMoa1300615 23991661

[7] Duraes AR, . Rivaroxaban versus warfarin in patients with mechanical heart valves: open-label, proof-of-concept trial—the RIWA study. Am J Cardiovasc Drugs. 2021;21(3):363-371. 33150497 10.1007/s40256-020-00449-3

[8] Carvalho Silva DM, Braga A, de Jesus I, Neves J. Mechanical prosthetic heart valve thrombosis in a patient receiving rivaroxaban. Cardiology. 2019;143(3-4):116-120. doi:10.1159/000501361 31473736

[9] Kumar V, Kelly S, Raizada A, . Mechanical valve thrombosis on rivaroxaban: are novel anticoagulants really an option? Methodist Debakey Cardiovasc J. 2017;13(2):73-75. doi:10.14797/mdcj-13-2-73 28740586 PMC5512683

[10] Lester PA, Coleman DM, Diaz JA, . Apixaban versus warfarin for mechanical heart valve thromboprophylaxis in a swine aortic heterotopic valve model. Arterioscler Thromb Vasc Biol. 2017;37(5):942-948. doi:10.1161/ATVBAHA.116.308649 28232327

[11] Shen AY, Yao JF, Brar SS, Jorgensen MB, Chen W. Racial/ethnic differences in the risk of intracranial hemorrhage among patients with atrial fibrillation. J Am Coll Cardiol. 2007;50(4):309-315. doi:10.1016/j.jacc.2007.01.098 17659197

[12] Higashi MK, Veenstra DL, Kondo LM, . Association between CYP2C9 genetic variants and anticoagulation-related outcomes during warfarin therapy. JAMA. 2002;287(13):1690-1698. doi:10.1001/jama.287.13.1690 11926893

[13] Limdi NA, Brown TM, Yan Q, . Race influences warfarin dose changes associated with genetic factors. Blood. 2015;126(4):539-545. doi:10.1182/blood-2015-02-627042 26024874 PMC4513254

[14] Tsai MS, Lin MH, Lee CP, . Chang Gung Research Database: a multi-institutional database consisting of original medical records. Biomed J. 2017;40(5):263-269. doi:10.1016/j.bj.2017.08.002 29179881 PMC6138604

[15] You JH, Chan FW, Wong RS, Cheng G. Is INR between 2.0 and 3.0 the optimal level for Chinese patients on warfarin therapy for moderate-intensity anticoagulation? Br J Clin Pharmacol. 2005;59(5):582-587. doi:10.1111/j.1365-2125.2005.02361.x 15842557 PMC1884850

[16] Loeliger EA, Broekmans AW. Optimal therapeutic anticoagulation. Haemostasis. 1985;15(4):283-292. 4043830 10.1159/000215161

[17] Gohlke-Bärwolf C, Acar J, Oakley C, ; Study Group of the Working Group on Valvular Heart Disease of the European Society of Cardiology. Guidelines for prevention of thromboembolic events in valvular heart disease. Eur Heart J. 1995;16(10):1320-1330. doi:10.1093/oxfordjournals.eurheartj.a060739 8746900

[18] Acar J, Iung B, Boissel JP, . AREVA: multicenter randomized comparison of low-dose versus standard-dose anticoagulation in patients with mechanical prosthetic heart valves. Circulation. 1996;94(9):2107-2112. doi:10.1161/01.CIR.94.9.2107 8901659

[19] Torella M, Torella D, Chiodini P, . Lowering the intensity of oral anticoagulant therapy in patients with bileaflet mechanical aortic valve replacement: results from the “LOWERING-IT” Trial. Am Heart J. 2010;160(1):171-178. doi:10.1016/j.ahj.2010.05.005 20598989

[20] Pruefer D, Dahm M, Dohmen G, Horstkotte D, Bergemann R, Oelert H. Intensity of oral anticoagulation after implantation of St Jude Medical mitral or multiple valve replacement: lessons learned from GELIA (GELIA 5). Eur Heart J Suppl. 2001; 3(suppl_Q):Q39-Q43. doi:10.1016/S1520-765X(01)90041-0

[21] Hering D, Piper C, Bergemann R, . Thromboembolic and bleeding complications following St. Jude Medical valve replacement: results of the German Experience With Low-Intensity Anticoagulation Study. Chest. 2005;127(1):53-59. doi:10.1378/chest.127.1.53 15653962

[22] Inoue H, Okumura K, Atarashi H, ; J-RHYTHM Registry Investigators. Target international normalized ratio values for preventing thromboembolic and hemorrhagic events in Japanese patients with non-valvular atrial fibrillation: results of the J-RHYTHM Registry. Circ J. 2013;77(9):2264-2270. doi:10.1253/circj.CJ-13-0290 23708863

[23] Cheung CM, Tsoi TH, Huang CY. The lowest effective intensity of prophylactic anticoagulation for patients with atrial fibrillation. Cerebrovasc Dis. 2005;20(2):114-119. doi:10.1159/000086801 16006759

[24] Liu T, Hui J, Hou YY, . Meta-analysis of efficacy and safety of low-intensity warfarin therapy for East Asian patients with nonvalvular atrial fibrillation. Am J Cardiol. 2017;120(9):1562-1567. doi:10.1016/j.amjcard.2017.07.050 28847595

[25] Mori T, Asano M, Ohtake H, . Anticoagulant therapy after prosthetic valve replacement-optimal PT-INR in Japanese patients-. Ann Thorac Cardiovasc Surg. 2002;8(2):83-87. 12027793

[26] Yu HY, Ho YL, Chu SH, Chen YS, Wang SS, Lin FY. Long-term evaluation of Carpentier-Edwards porcine bioprosthesis for rheumatic heart disease. J Thorac Cardiovasc Surg. 2003;126(1):80-89. doi:10.1016/S0022-5223(02)73608-8 12878942

[27] Cheng CL, Lee CH, Chen PS, Li YH, Lin SJ, Yang YH. Validation of acute myocardial infarction cases in the national health insurance research database in Taiwan. J Epidemiol. 2014;24(6):500-507. doi:10.2188/jea.JE20140076 25174915 PMC4213225

[28] Hsieh CY, Chen CH, Li CY, Lai ML. Validating the diagnosis of acute ischemic stroke in a National Health Insurance claims database. J Formos Med Assoc. 2015;114(3):254-259. doi:10.1016/j.jfma.2013.09.009 24140108

[29] Hung LC, Sung SF, Hsieh CY, . Validation of a novel claims-based stroke severity index in patients with intracerebral hemorrhage. J Epidemiol. 2017;27(1):24-29. doi:10.1016/j.je.2016.08.003 28135194 PMC5328736

[30] Puskas J, Gerdisch M, Nichols D, ; PROACT Investigators. Reduced anticoagulation after mechanical aortic valve replacement: interim results from the prospective randomized on-X valve anticoagulation clinical trial randomized Food and Drug Administration investigational device exemption trial. J Thorac Cardiovasc Surg. 2014;147(4):1202-1210. doi:10.1016/j.jtcvs.2014.01.004 24512654

