# Supplementary material for: Analysis of Anticoagulation Therapy and Anticoagulation-Related Outcomes Among Asian Patients After Mechanical Valve Replacement
Source: JAMA Netw Open. 2022 Feb 1;5(2):e2146026. doi: 10.1001/jamanetworkopen.2021.46026 (PMC8808330; doi:10.1001/jamanetworkopen.2021.46026)
Supplement: Supplement. — eFigure. Average INR Value Across 2001 to 2018 in Patients With Isolated Aortic Valve Replacement and Mitral Valve Replacement or Double Valve Replacement eTable 1. Association Between INR Value and Risk of Thromboembolic and Bleeding Events in Patients Undergoing AVR Surgery eTable 2. Association Between INR and Risks of Thromboembolic and Bleeding Events in Patients Receiving MVR Alone or MVR-AVR Combination Surgery [file jamanetwopen-e2146026-s001.pdf]

Supplemental Online Content

Huang JT, Chan YH, Wu VCC, et al. Analysis of anticoagulation therapy and anticoagulation-related outcomes among Asian patients after mechanical valve replacement. *JAMA Netw Open.* 2022;5(2):e2146026. doi:10.1001/jamanetworkopen.2021.46026

eFigure. Average INR Value Across 2001 to 2018 in Patients With Isolated Aortic Valve Replacement and Mitral Valve Replacement or Double Valve Replacement

eTable 1. Association Between INR Value and Risk of Thromboembolic and Bleeding Events in Patients Undergoing AVR Surgery

eTable 2. Association Between INR and Risks of Thromboembolic and Bleeding Events in Patients Receiving MVR Alone or MVR-AVR Combination Surgery

This supplemental material has been provided by the authors to give readers additional information about their work.

© 2022 Huang JT et al. *JAMA Network Open.*

eFigure. Average INR Value Across 2001 to 2018 in Patients With Isolated Aortic Valve Replacement and Mitral Valve Replacement or Double Valve Replacement

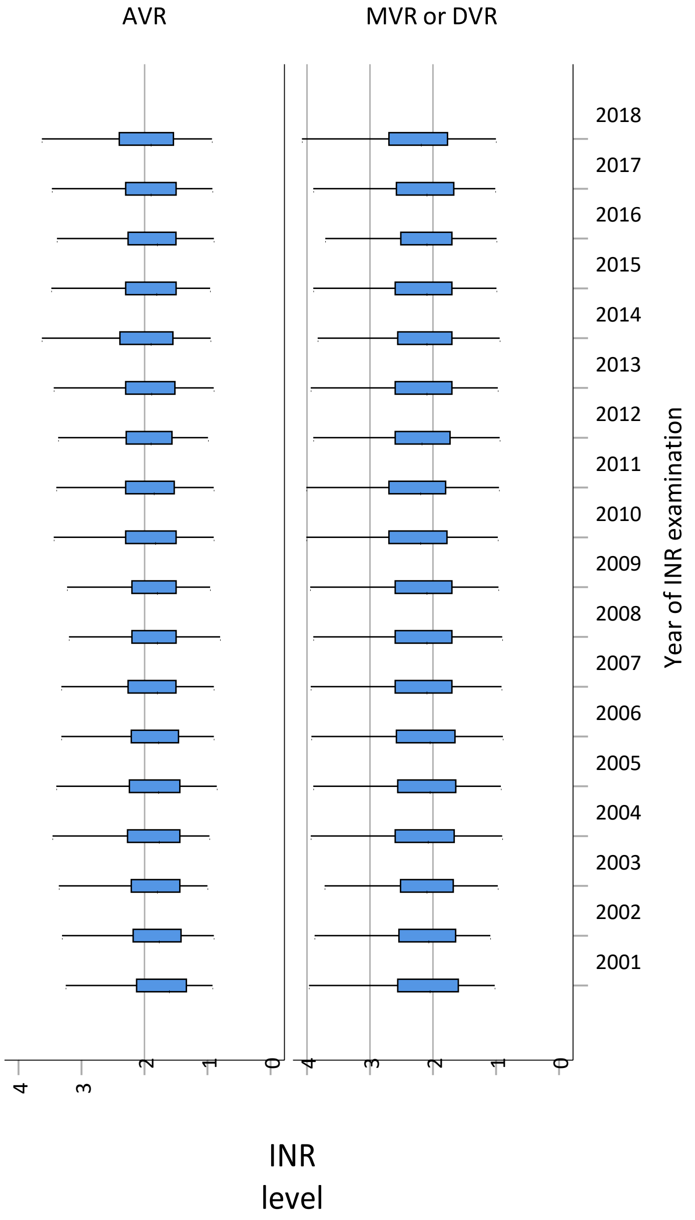

© 2022 Huang JT et al. *JAMA Network Open.*

eTable 1. Association Between INR Value and Risk of Thromboembolic and Bleeding Events in Patients Undergoing AVR Surgery

	Any thromboembolic event					Any bleeding event			
Outcome	Number of observation	No. of event (%)	OR (95% CI)	aOR (95% CI)		Number of observation	No. of event (%)	OR (95% CI)	aOR (95% CI)
Continuous exposure for INR	-	-	0.65 (0.33–1.28)	0.77 (0.46–1.27)		-	-	1.71 (1.48–1.96)*	1.62 (1.36–1.92)*
Categorical exposure for INR									
<1.5	3,818	45 (1.2)	3.55 (1.86–6.80)*	2.55 (1.37–4.73)*		3,738	54 (1.4)	1.25 (0.77–2.03)	1.14 (0.66–1.98)
1.5-2	6,085	24 (0.39)	1.28 (0.64–2.54)	1.15 (0.55–2.41)		6,730	49 (0.73)	0.77 (0.49–1.21)	0.87 (0.48–1.56)
2-2.5	4,185	14 (0.33)	Reference	Reference		3,540	36 (1.0)	Reference	Reference
2.5-3	1,539	7 (0.45)	1.21 (0.42–3.42)	0.96 (0.33–2.75)		1,539	20 (1.3)	1.21 (0.67–2.17)	1.36 (0.72–2.57)
≥3	1,049	8 (0.46)	1.97 (0.73–5.33)	1.70 (0.67–4.30)		1,129	52 (4.6)	4.09 (2.52–6.63)*	3.48 (1.95–6.23)*

Abbreviations: INR, international normalized ratio; AVR, aorta valve replacement; OR, odds ratio; aOR, adjusted odds ratio; CI, confidence interval.

‡The model was adjusted for all covariates listed in Table 1.

*$P < 0.05$.

© 2022 Huang JT et al. *JAMA Network Open.*

eTable 2. Association Between INR and Risks of Thromboembolic and Bleeding Events in Patients Receiving MVR Alone or MVR-AVR Combination Surgery

	Any thromboembolic event					Any bleeding event			
Outcome	Number of observation	No. of event (%)	OR (95% CI)	aOR (95% CI)		Number of observation	No. of event (%)	OR (95% CI)	aOR (95% CI)
Continuous exposure for INR	-	-	0.81 (0.49–1.35)	0.91 (0.57–1.44)		-	-	1.39 (1.23–1.57)	1.36 (1.19–1.55)
Categorical exposure for INR									
<2	2676	31 (1.2)	2.04 (0.95–4.38)	1.68 (0.68–4.18)		2520	48 (1.9)	0.98 (0.66–1.46)	0.79 (0.50–1.25)
2-2.5	4943	18 (0.36)	1.34 (0.54–3.31)	1.19 (0.39–3.58)		5545	41 (0.74)	0.69 (0.42–1.13)	0.59 (0.33–1.08)
2.5-3	5615	23 (0.41)	Reference	Reference		5013	33 (0.66)	Reference	Reference
3-3.5	2697	9 (0.33)	1.74 (0.58–5.25)	1.41 (0.36–5.54)		2697	27 (1.0)	1.43 (0.86–2.38)	1.40 (0.79–2.46)
≥3.5	2276	15 (0.66)	2.39 (0.91–6.28)	2.19 (0.83–5.74)		2342	61 (2.5)	2.96 (1.90–4.60)*	2.25 (1.35–3.76)*

Abbreviations: INR, international normalized ratio; MVR, mitral valve replacement; OR, odds ratio; aOR, adjusted odds ratio; CI, confidence interval.

‡The model was adjusted for all covariates listed in Table 1.

*P < 0.05.

© 2022 Huang JT et al. *JAMA Network Open.*
